# Molecular Cloning, Characterization and Expression of the Phenylalanine Ammonia-Lyase Gene from *Juglans regia*

**DOI:** 10.3390/molecules17077810

**Published:** 2012-06-26

**Authors:** Feng Xu, Guang Deng, Shuiyuan Cheng, Weiwei Zhang, Xiaohua Huang, Linling Li, Hua Cheng, Xiaofeng Rong, Jinbao Li

**Affiliations:** 1 National Engineering Laboratory for Tree Breeding, Key Laboratory of Genetics and Breeding in Forest Trees and Ornamental Plants, Ministry of Education, Beijing Forestry University, Beijing 100083, China; 2 College of Horticulture and Gardening, Yangtze University, Jingzhou 434025, Hubei, China; 3 College of Chemistry and Molecular Engineering, Peking University, Beijing 100871, China; 4 Hubei Key Laboratory of Economic Forest Germplasm Improvement and Resources Comprehensive Utilization, Huanggang Normal University, Huanggang 438000, Hubei, China

**Keywords:** phenylalanine ammonia-lyase (PAL), *Juglans regia*, molecular cloning, expression pattern

## Abstract

Phenylalanine ammonia-lyase (PAL) is the first key enzyme of the phenypropanoid pathway. A full-length cDNA of *PAL* gene was isolated from *Juglans regia* for the first time, and designated as *JrPAL*. The full-length cDNA of the *JrPAL* gene contained a 1935bp open reading frame encoding a 645-amino-acid protein with a calculated molecular weight of about 70.4 kD and isoelectric point (pI) of 6.7. The deduced JrPAL protein showed high identities with other plant PALs. Molecular modeling of JrPAL showed that the 3D model of JrPAL was similar to that of PAL protein from *Petroselinum crispum* (PcPAL), implying that JrPAL may have similar functions with PcPAL. Phylogenetic tree analysis revealed that JrPAL shared the same evolutionary ancestor of other PALs and had a closer relationship with other angiosperm species. Transcription analysis revealed that *JrPAL* was expressed in all tested tissues including roots, stems, and leaves, with the highest transcription level being found in roots. Expression profiling analyses by real-time PCR revealed that *JrPAL* expression was induced by a variety of abiotic and biotic stresses, including UV-B, wounding, cold, abscisic acid and salicylic acid.

## 1. Introduction

Phenylalanine ammonia-lyase (PAL, EC 4.3.1.5) catalyzes the deamination of phenylalanine to *trans*-cinnamic acid and ammonia, the first step in the biosynthesis of phenylpropanoids [1,2]. Many secondary metabolic products in plants, such as anthocyanins, flavonoids, ultraviolet (UV) protectants, antimicrobial furanocoumarins, isoflavonoid phytoalexins, lignins and wound phenolic esters are phenylpropanoid derivatives [3,4,5]. The production of phenylpropanoid compounds is important in plant development, plant-microbe signalling and plant defence [3,6]. The PAL enzyme has been considered to be the one of the key enzymes in the biosynthesis of flavonoids [3,7], due to the central function of PAL as the first enzyme in phenylpropanoid derivative metabolism, and PAL is also a key enzyme in plant stress response. The expression of PAL isozymes can be induced by tissue wounding, plant growth regulators, UV irradiation, low temperature, low levels of nitrogen, phosphate, and iron [8,9,10,11]. Along with the research on flavonoid metabolic pathways, many intermediates are found to possess certain positive roles in different tissues, such as UV protection, resistances against pathogens and insects, which is required for plant defense system and plant systemic resistance [12,13]. Thus, the study on the characterization and expression pattern of the genes involved in the flavonoid biosynthetic pathway, in particular the well-documented genes such as *PAL*, is essential to further understanding the mechanism of stress resistance and the biosynthesis of flavonoids [14].

*PAL* is present in all higher plants studied and is also found in some fungi [15,16], cyanobacteria [17], and *Streptomyces* [18]. Because of its crucial role in the biosynthesis of flavonoids, lignins, and phytoalexins and stress responses, PAL and its gene are widely studied [3,19]. So far, *PAL* have been isolated from different species of plants and studied at the molecular level. *PAL* genes in some species such as *Petroselinum crispum* [20], *Arabidopsis thaliana* [21], *Salvia miltiorrhiza* [22], and *Solanum lycopersicum* [23] belong to a small gene family, and PAL is encoded by a multi-gene family. Each member of the PAL family showed a distinctive expression pattern, and some PAL isoforms have bifunctional enzymatic activities that can modulate different metabolic pathways [21,24]. Several studies have shown that PAL from monocot plants also has tyrosine ammonia-lyase activity [25,26].

*Juglans regia* L. from the Juglandaceae family, is widely distributed all over the world and common in China. *J. regia* is not only an agricultural commodity, but its leaves, barks, stems, pericarps, fruits, flowers and ligneous membranes are all applied for different medicinal use in China. These fruits are attracting increasing interest as a healthy foodstuff because their regular consumption has been reported to decrease the risk of coronary heart disease [27,28,29]. The accumulation of flavonoids, as important bioactive constituents of *J. regia*, requires coordinated expression of genes encoding enzymes in the core phenylpropanoid pathway, such as PAL and 4-coumarate CoA ligase, and enzymes in branch pathways such as chalcone synthase and chalcone isomerase. However, to the best of our knowledge, there has been no report on cloning and expression profiles of *PAL* gene in *J. regia*. In order to confirm the function of *PAL* in *J. regia*, the full-length cDNA sequence of *PAL* gene were isolated and characterized from *J. regia* for the first time. The three-dimensional structural model, the phylogenetic trees, and expression profiles of *JrPAL* in different tissues and under stresses were also investigated.

## 2. Results and Discussion

### 2.1. Characterization of the Full-Length cDNA Sequence of JrPAL Gene and Deduced JrPAL Protein

**Figure 1:**
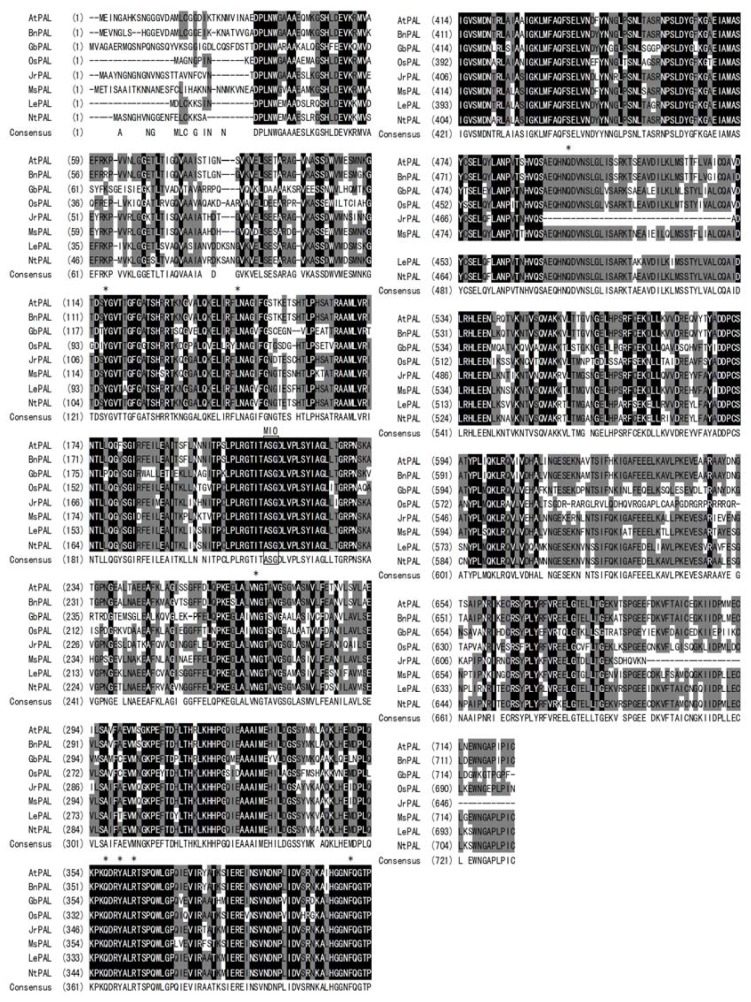
Sequence multialignment of the deduced JrPAL protein with other PALs. The completely identical amino acids are indicated with white foreground and black background. The conserved amino acids are indicated with black foreground and background. Non-similar amino acids are indicated with black foreground and white background. The active sites residues are indicated in asterisk (*), and residues Ala-Ser-Gly forming MIO group are boxed in the alignment. AtPAL, PAL from *A. thaliana* (NP181241); BnPAL, PAL from *Brassica napus* (ABC69916); GbPAL, PAL from *Ginkgo biloba* (ABU49842); OsPAL, PAL from *Oryza sativa* (CAA34226); MsPAL, PAL from *Medicago sativa* (CAA41169); LePAL, PAL from *Lycopersicon esulentum* (AAA34179); NtPAL, PAL from *N. tabacum* (BAA22948).

Using a RACE-PCR method, the full-length cDNA sequence of *PAL* gene was finally obtained from *J. regia* (designated as *JrPAL*, GenBank accession no. JX069977). It had 2267 bp with a poly (A) tail, and contained a 1935 bp open reading frame (ORF) encoding a 645 amino acid protein. There was a 5′ untranslated region of 135 bp upstream from the start codon, and the coding region was followed by a 3′ untranslated region that was 194 bp long downstream from the stop codon. By using the computer software pI/Mw Tool at ExPASy, the calculated molecular weight and isoelectric point (pI) of the deduced JrPAL protein were predicted to be 70.4 kD and 6.7, respectively. Blast in NCBI and multi-alignment by Vector NTI 10.0 revealed that the deduced JrPAL had considerable homology to other plant PALs. It was found that JrPAL presented 83% identity to both *Lycopersicon esulentum* and *Nicotiana tabacum*, 82% identity to *Medicago sativa*, and 81% identity to both *A. thaliana* and *Brassica napus*. JrPAL also showed 68% identities to Poaceae plants as *Oryza sativa* and 66% identities to Gymnosperms like *Ginkgo biloba*. A high similarity among PAL proteins was observed from residues 35 to 692, with variability in length and composition being found in the N-terminal and C-terminal regions (Figure 1). Many sites essential to PAL activities conserved in different plant species were also found in JrPAL (Figure 1). For example, active site amino-acid residues in JrPAL were S204, the predominant precursor of 4-methylidene-imidazole-5-one (MIO) group, as well as Y109, L197, N261, Q349, Y352, R355 and F401. These active site residues may be important for substrate binding, catalysis or MIO formation. It has been proved that a prosthetic MIO group was formed autocatalyticallly by cyclization and dehydration of an Ala-Ser-Gly tripeptide, representing highly conserved residues with the PAL and HAL protein families [14,30]. All the active sites mentioned above were the same and at the counterpart positions with those of PAL from *Petroselinum crispum* [28], indicating JrPAL was a member of PAL family. The multi-alignment results of JrPAL with other plant PAL protein sequences revealed that about 41 amino acid residues was absented in JrPAL including an active site Q, which was position at about 483 in other plant PAL protein sequences.

### 2.2. Three-Dimensional Model Analysis

**Figure 2:**
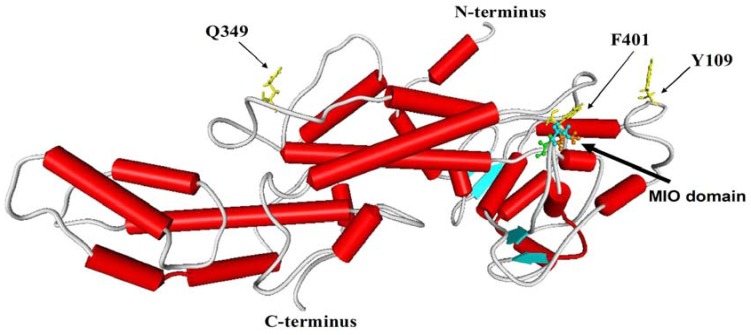
The 3D structure of JrPAL established by homology-based modeling. The helix, sheet and random coil are indicated by the column, arrow plate and rope shape, respectively. The MIO and active site residues of JrPAL are shown.

In order to better understand the deduced JrPAL protein, a comparative modeling of 3D model of JrPAL was performed at ExPASy using SWISS-MODEL [29]. 3-D structural alignment and analyses were performed using Weblab Viewerlite 3.5, and the template for modeling was 3D structure of PcPAL (PDB No.1w27A). As it can be seen from Figure 2, the model covered the 26-638 amino acids of JrPAL and consisted of α-helices (52.56%), β-turns and random coils (37.53%), and extended strands (9.92%). The MIO domain shown on the top contained the Ala-Ser-Gly tripeptide, which acts as active site.

### 2.3. Molecular Evolution Analysis

**Figure 3:**
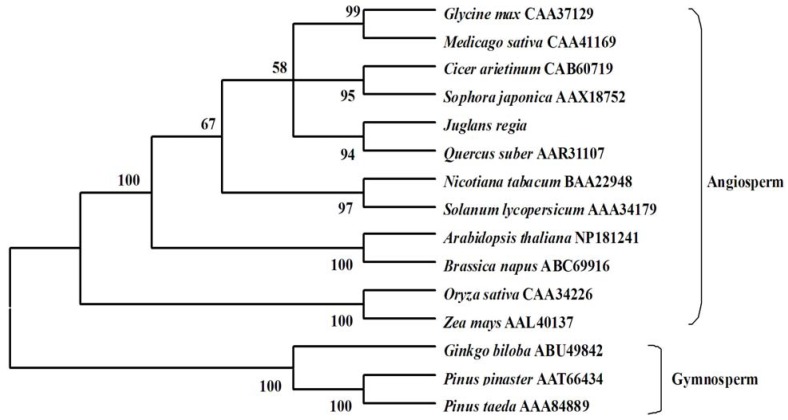
The phylogenetic analysis of PALs from *J. regia* and other plant species by MEGA 4 from CLUSTAL W alignments. The neighbor-joining method was used to construct the tree with p-distance. The number for each interior branch is the percent bootstraps value (100 replicates).

To investigate the evolutionary relationships among the deduced JrPAL and PALs from other plant species, the phylogenetic tree was constructed using a neighbor-joining method. As reported in Figure 3, the phylogenetic tree was grouped into two main branches, including angiosperm species and gymnosperm species. The result showed that *J. regia* was clustered with another Fagaceae species, *Quercus suber*, indicating that *J. regia* has a closer relationship with angiosperm species than gymnosperm species. It was also obvious that the phylogenetic tree was grouped in a family-specific manner. For example, two members of Solanaceae PALs from *Solanum lycopersicum* and *N. tabacum* had a closer relationship than others. The two Poaceae species *Zea mays* and *Oryza sativa* were grouped into a cluster, while *A. thaliana* and *Brassica napus* from the Cruciferae were grouped into a cluster. As could be seen from the tree, *Cicer arietinum* and *Sophora japonica* formed a cluster, as did *Glycine max* and *Medicago sativa*, all species belonging to theFagaceae family. Our results suggested that JrPAL shared a common evolutionary ancestor with other PALs based on conserved structure and sequence characteristics such as amino acid homologies and conserved motifs, and *J*. *regia* had a further relationship with those gymnosperm species.

### 2.4. Transcript Accumulation of JrPAL in Different Tissues and Under Different Stresses

**Figure 4:**
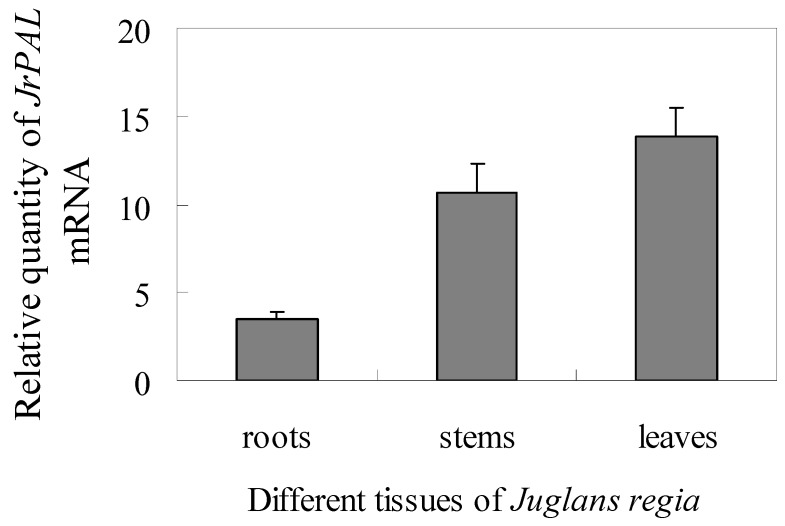
Expression pattern of *JrPAL* in different tissues of *J. regia* including leaves, stems and roots. Each tissue sample was individually assayed in triplicate. Values shown represent the mean reading from three independent analyses and the error bars indicate the standard error of the mean.

To investigate the expression pattern of *JrPAL* gene in different tissues of *J. regia*, total RNA was isolated from roots, stems and leaves respectively, and used for real-time PCR analysis. The results showed that the *JrPAL* transcripts could be detected in all of the tested tissues, and with the highest transcript levels being found in root (Figure 4). The *JrPAL* transcript level in leaves is the lowest, about one third that of stems and a quarter of that of roots. The *PAL* transcript accumulation data in *J. regia* was consistent with other studies showing that *PAL* mRNA was typically high in roots and low in leaves of other plants, such as *A. thaliana* [33], *Hordeum vulagare* [34] and *Solanum tuberosum* [35]. The high transcript levels in roots compared with other plant tissues may be due to the high rate of lignification, which is part of normal root development [36].

**Figure 5:**
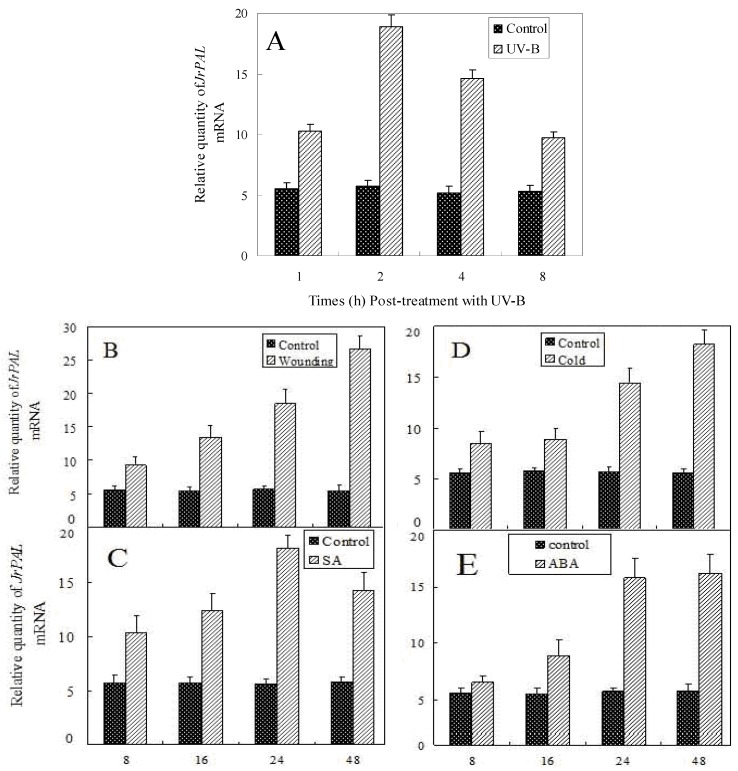
Relative quantities of *JrPAL* mRNA at various time points post-treatment with UV-B (**A**), wounding (**B**),SA (**C**), Cold (**D**) and ABA (**E**). Total RNA was isolated from the one-year old *J. regia* seedling leaves under the treatments of UV-B, wounding, cold, SA and ABA for various durations. The *J. regia 18S* gene was used as control. Each plant was individually assayed in triplicate. Values shown represent the mean reading from three plants and the error bars indicate the standard error of the means.

PAL activity could be induced by different abiotic stresses. In order to identify if the expression of *JrPAL* was also affected by these stresses, we detected the expression of *JrPAL* in plants exposed to different stressors using real-time PCR. The seedlings of *J. regia* were treated with UV-B, wounding, cold, SA and ABA for various durations and afterwards leaves were harvested for total RNA extraction. As shown in Figure 5, under UV-B treatment, *JrPAL* transcript accumulation significantly improved at 1 h and reached the highest level, about three times as much as controls, at 2 h. *JrPAL* transcript accumulation gradually declined after 2 h and reached the lowest level 8 h after UV-B treatment, but still showed a higher level than that of the control (0 h). The experimental results showed that wounding, cold, SA and ABA can also improve the expression level of *JrPAL* gene. Under wounding, cold and ABA treatments, the transcript accumulation increased along with the treatment time and reached the highest level at the end of treatment (48 h). Expression of *JrPAL* under SA treatments also gradually increased in the initial 24 h and then decreased slightly, but also still showed higher level than control (0 h). There was no significant different expression level of *JrPAL* in the initial 16 h with cold treatment, and no significant different expression level between 24 h and 48 h with abscisic acid treatment. These results were similar to those observed in leaves of barley and rice seedlings under UV-B irradiation [9,34], leaves of wounded Romaine lettuce [1], leaves of tetraploid *I. indigotica* under cold stress [37], tobacco and parsley leaves sprayed with SA [38,39], and ABA-treated fruit [40]. UV-B and wounding induce PAL activity and this is associated with the accumulation of phenylpropanoid products, which might protect the plant from UV damage and wounding [9]. The biotic elicitor SA exhibits a notable effect on the production of secondary metabolites such as phenylpropanoids and alkaloids [41] and it is considered as one of the key endogenous signals involved in the activation of numerous plant defense responses [32]. Because PAL is a key enzyme of the phenylpropanoid pathway, expression of *JrPAL* induced by SA could be considered as a part of the plant defense mechanisms [4]. The *JrPAL* could be markedly induced by cold treatment and the activation of phenylpropanoid metabolism may play a role in the development of protective barriers in cold-damaged cells [43]. ABA enhanced PAL activity in present study, similar to ABA increases in PAL activity and anthocyanin and phenolics levels in strawberry cultivars [44,45].

## 3. Experimental

### 3.1. Plant Materials and Treatments

One-year old seedlings of *J. regia* were grown in a greenhouse at Yangtze University, Jingzhou, China. The edges of *Juglans* leaves were cut into pieces of about 1 cm with scissors for wounding treatment. For UV-B treatment, the seedlings were exposed in a dark closed chamber to 1500 μJ/m^2^ UV-B irradiation, and the control seedlings were placed in a dark closed chamber. The cold treatment was performed by placing the seedlings in a 4 °C growth room, whereas the control was treated at 25 °C. Salicylic acid (SA) and abscisic acid (ABA) treatments were performed by spraying 500 μM SA and 100 μM ABA, respectively, and control plants were sprayed with the equal amount of distilled water. Leaves from the treated and control plants were harvested and immediately frozen in liquid nitrogen followed by storage at 80 °C until use.

### 3.2. DNA and RNA Extraction

Genomic DNA was extracted from the fresh leaves of *J. regia* following the CTAB method described by Zhang and Wan [46]. Total RNA was extracted separately from all samples by using CTAB method [47]. The quality and concentration of the RNA and genomic DNA were all determined by agarose gel electrophoresis and spectrophotometer analysis.

### 3.3. Cloning of the Conserved Fragment of JrPAL

To amplify conserved fragment of *JrPAL*, RT-PCR was performed using degenerate oligonucleotide primers designed to conserved amino acid regions in other plant species obtained from GenBank database using NCBI Blast. Degenerate forward and reverse primers used for *PAL* were respectively: PALsP, PALaP (Table 1). A length of the fragment was 866 bp obtained by using one step RT-PCR kit (TaKaRa) under the following PCR program: 50 °C for 30 min and 94 °C for 3 min, followed by 35 cycles of amplification (94 °C for 1 min, 50 °C for 1 min, 72 °C for 1 min). The PCR product was purified and cloned into the pMD18-T vector (Dalian TaKaRa, China), and then sequenced. Subsequent BLAST results confirmed that the amplified product was a partial fragment of the *PAL* gene.

**Table 1:** Primers used in molecular cloning and expression of *PAL* gene.

Primer Name	Primer Sequence
PALsP	5′-GCHTCBGGTGATYTRGTY-3′
PALaP	5′-ACATCTTGGTTRTGYGCTC-3′
JuPAL3	5′-CATCGGGCGACCTGGTTCCTCTGTCTTAC-3′
JuPAL3N	5′-GGCTGTAGGACCCAATGGAGAGT-3′
JuPAL5	5′-GTCGCAGAGTGAGGTAATGTGTGGCAAGA-3′
JuPAL5N	5′-CCATTGCCAAAGATTCCAGAGTT-3′
JrPALUTR	5′-TCCAAGTTTTCCTTTGCGTTT-3′
PALRT-S	5′-CTGGGATCAATGGTGGGTTT-3′
PALRT-A	5′-TATCGGTCTTGTTTGGGCTT-3′
PAL prob	5′-GAGTTACAACCCAAAGAAG-3′
18S-S	5′-GGTCAATCTTCTCGTTCCCTT-3′
18S-A	5′-TCGCATTTCGCTACGTTCTT-3′
18S prob	5′-AAACAAGGAGTAACCACGG-3′

### 3.4. 3′RACE and 5′RACE of JrPAL Gene

Based on the sequence of the cloned internal conservative fragment of the *JrPAL* gene, the four specific primers, JrPAL3 and JrPAL5 (Table 1), as well as the nested primers JrPAL3N and JrPAL5N (Table 1) were designed and synthesized to amplify the 3′ and 5′ ends of the *JrPAL* gene using the SMART^TM^ RACE cDNA Amplification kit (Clontech, CA, USA). An aliquot of total RNA (1 µg) was reversely transcribed to get the 3′-ready cDNA with 3′-CDS primer A (provided in the kit). Universal Primer A Mix (UPM, provided in the kit), JrPAL3 and 3′-ready cDNA were used for the first round of 3′ RACE, the reaction was performed at 94 °C for 3 min, then 94 °C for 30 s and 70 °C for 3 min with 5 cycles, followed by 94 °C for 30 s, 68 °C for 30 s and 72 °C for 3 min with 30 cycles, then 72 °C for 10 min. Subsequently, JrPAL3N and Nested Universal Primer A (NUP, provided in the kit) were used for the nested PCR amplification, the reaction was performed at 94 °C for 3 min, followed by 94 °C for 30 s, 68 °C for 30 s and 72 °C for 3 min with 35 cycles, then 72 °C for 10 min. The nested PCR amplified product was purified and cloned into pMD18-T vector followed by sequencing.

The 5′-ready cDNA was synthesized with 5′-CDS primer A and SMART II A oligonucleotide (provided in the kit). The first round of PCR was performed with primers JrPAL5 and UPM under the following condition: 94 °C for 3 min, then 94 °C for 30 s and 70 °C for 3 min with 5 cycles, followed by 94 °C for 30 s, 70 °C for 30 s, and 72 °C for 3 min with 5 cycles, then 94 °C for 30 s, 68 °C for 30 s, 72 °C for 3 min with 25 cycles, and finally 72 °C for 10 min. The PCR product was then used for the nested PCR amplification with primers JrPAL5N and NUP, the reactions was performed at 94 °C for 3 min followed by 94 °C for 30 s, 68 °C for 30 s and 72 °C for 3 min with 35 cycles, then 72 °C for 10 min. The nested PCR amplified product was purified and cloned into pMD18-T vector followed by sequencing.

### 3.5. Generation of the Full-Length cDNA of JrPAL

After comparing and aligning the sequences of the 5′RACE, 3′RACE, and the internal region products, the full length cDNA sequence of *JrPAL* was obtained through RT-PCR with JrPALUTR (Table 1) and UPM (provided in the kit) as primers, and 3′-ready cDNA library as template by using One Step RT-PCR Kit (Takara, Dalian, China) under the following conditions: 94 °C for 3 min followed by 35 cycles of amplification (94 °C for 20 s, 68 °C for 30 s and 72 °C for 2 min). The PCR product was purified and cloned into pMD18-T vector followed by sequencing. The full-length cDNA of *JrPAL* was subsequently analyzed for molecular characterization after sequencing.

### 3.6. Bioinformatic and Molecular Evolution Analyses

The obtained sequences were analyzed using online bioinformatics tools from NCBI and ExPASy. The software Vector NTI Advance 10 was used for sequence multialignment. The deduced JrPAL and other known PAL sequences retrieved from GenBank were aligned with CLUSTAL. Subsequently, a phylogenetic tree was constructed using neighbour-joining (NJ) method with MEGA 4 software [48,49,50]. The reliability of the tree was measured by bootstrap analysis with 1,000 replicates [51].

### 3.7. Quantification of Transcript Levels by Real-Time PCR Assay

Real-time PCR was used to investigate the transcription levels of *JrPAL* in the different *J. regia* tissues and in the treated leaf samples from the young seedlings collected at different post- treatment time points Real-time PCR was performed in the Perkin-Elmer 7000 thermal cycler according to the manufacturer’s protocol. Total RNA was isolated from *J. regia* for each plant sample and treated with Rnase-free Dnase I at 37 °C for 30 min using the Dnase I kit (TaKaRa, Dalian, China). The primers and TaqMan MGB probes for *JrPAL* (PALRT-S, PALRT-A and PAL prob) and the house-keeping gene *18S* (18S-S, 18S-A and 18S prob) were designed using the Sequence Detection System software, and are shown in Table 1, respectively. The primers and probes were synthesized and obtained from Real-Time Laboratory (Dalian TaKaRa). The primers and the TaqMan MGB probe of 18S were used at a final concentration of 500 nM and 250 nM, respectively, while the primers and the TaqMan MGB probe of *JrPAL* were used at a final concentration of 1 µM and 100 nM, respectively. For each plant sample, aliquots of total RNA (200 ng) were analyzed for each gene. The PCR used one-step RT-PCR master mix reagents (Dalian TaKaRa), a reaction mixture (50 µL) was set up in a 96-well plate. The two genes were always analyzed simultaneously. Each sample was run in triplicate. The real-time PCR conditions were: 30 min at 48 °C, and 40 cycles of 15s at 95 °C and 1 min at 60 °C. With housekeeping gene 18S, the relative amount of the *JrPAL* transcript in the different tissues was obtained according to the comparative threshold cycle (*C_T_*) method described in the Real-Time PCR Applications Guide (Perkin-Elmer User Bulletin 2). For the post-abiotic treatment analysis of *JrPAL* expression at different time points, the induction level of *JrPAL* was obtained according to the transcription levels of *JrPAL* in the treated plants compared with those of the control plants. The real-time PCR with no template mRNA control was employed as minus RT controls.

### 3.8. Statistical Analyses

The data determined in triplicate were analyzed by analysis of variance (ANOVA) using SPSS (version 16.0). The significance of differences was determined according to Duncan’s multiple range test (DMRT). *P* values < 0.05 are considered to be significant.

## 4. Conclusions

In the present study, we successfully isolated and characterized a *PAL* gene from *J. regia* and analyzed its expression profiles in different tissues and under stresses. Relationship analysis based on the phylogenetic tree constructed on the basis of putative amino acid sequences demonstrated that *JrPAL* was most closely related to another Fagaceae species, *Q. suber*, among the surveyed plant species. Multiple alignments of amino acid sequences between JrPAL and others showed that the former also possessed many active sites that are well-conserved in different plant species. Moreover, JrPAL is missing about 41 amino acid residues, including an active site Q from the site 483, which might be caused by evolution or mRNA splicing. Our data revealed that transcript levels of the *JrPAL* gene were induced by different abiotic stresses, implying that *JrPAL* may be a stress-responsive gene. Therefore, *JrPAL* could be considered as a potential target gene to be used in genetic engineering for creation of transgenic plants with improved stress tolerance. However, it has to be pointed out that our work only provided data on expression of *JrPAL* under a single particular stress. For further understanding of the *JrPAL* and its function, a plant expression vector containing the *JrPAL* has been constructed and a study on the genetic transformation of *J. regia* is underway, in order to test its potential role in improving stress tolerance and flavonoid accumulation by genetic engineering.
